# Severe Iron Deficiency Anemia With Koilonychia and Endoscopically Detected Duodenal Ascariasis in an Elderly Male: A Case Report

**DOI:** 10.7759/cureus.117435

**Published:** 2026-10-04

**Authors:** Samiha Mobashshira Rotshi, Saptarsi Mondal, Subrata Kumar Biswas, Tasnova Jaman Liana

**Affiliations:** 1 Medicine, Dhaka Medical College and Hospital, Dhaka, BGD

**Keywords:** ascariasis, ascaris lumbricoides, iron deficiency anemia, koilonychia, stool microscopy, upper gastrointestinal endoscopy

## Abstract

Iron deficiency anemia is a common nutritional and clinical condition that can arise from chronic blood loss, insufficient dietary intake, impaired absorption, or parasitic infection, especially in areas where such infections are endemic. In elderly male patients, a thorough evaluation is necessary to rule out gastrointestinal bleeding and malignancy. A 60-year-old male farmer experienced exertional dyspnea, generalized weakness, pallor, weight loss, and gradual changes in his nails. Physical examination revealed the characteristic spoon-shaped nails of koilonychia. Laboratory tests confirmed severe microcytic hypochromic anemia with significantly depleted iron stores, as indicated by a hemoglobin level of 4.2 g/dL, serum ferritin of 1.92 ng/mL, low serum iron, and elevated total iron-binding capacity. Upper gastrointestinal endoscopy, performed to investigate the severe iron deficiency anemia, revealed multiple live *Ascaris lumbricoides* worms in the duodenum, consistent with duodenal ascariasis. The patient was treated with albendazole, red cell concentrate transfusion based on his clinical condition, and oral iron supplementation. At the three-month follow-up, he demonstrated marked clinical, hematological, and biochemical improvement, including improvement of koilonychia. This case underscores that parasitic infestation can coexist with or contribute to severe iron deficiency anemia in endemic areas. Stool microscopy remains an important standard diagnostic test, whereas endoscopy should be viewed as part of a broader evaluation for unexplained severe anemia, not as a first-line test for ascariasis.

## Introduction

Iron deficiency anemia is a frequently encountered clinical condition that can arise from various factors, such as chronic blood loss, insufficient dietary intake, decreased absorption, and elevated physiological requirements [1]. In adult and elderly male patients, iron deficiency anemia should not be solely attributed to nutritional deficiency without proper assessment, as gastrointestinal bleeding, malignancy, and malabsorption may be underlying etiologies [1].

Parasitic infections continue to hold clinical significance in endemic areas. Soil-transmitted helminths are linked to poor sanitation, unsafe water, and fecal contamination of soil, and the risk may be elevated in individuals with occupational exposure to contaminated soil [2,3]. Hookworm infection is the helminthic disease most typically associated with iron deficiency anemia because it can lead to chronic intestinal blood loss. Ascariasis is less directly connected to iron loss, but it may contribute to anemia through decreased intake, impaired absorption, nutritional competition, chronic intestinal disturbance, and coexistence with other parasitic or gastrointestinal conditions [4,5].

Koilonychia, or spoon-shaped nails, is a valuable clinical indicator of chronic iron deficiency anemia, though it is not specific. It has also been observed in cases of malnutrition, gastrointestinal blood loss, celiac disease, thyroid disease, repeated trauma, occupational exposure, cardiovascular disease, systemic lupus erythematosus, Raynaud phenomenon, and other conditions [6]. Therefore, koilonychia should lead to an investigation for chronic iron deficiency and its underlying cause rather than being considered a diagnosis in itself.

This case describes an elderly male farmer with severe iron deficiency anemia and koilonychia, in whom duodenal ascariasis was identified during upper gastrointestinal endoscopy. The aim of this report is not to advocate endoscopy as a first-line diagnostic method for ascariasis, but rather to emphasize a balanced diagnostic approach in endemic settings, where parasitic infection should be considered while gastrointestinal blood loss, malignancy, malabsorption, and other causes of iron deficiency anemia are also assessed.

## Case presentation

A 60-year-old male farmer from South Asia presented with shortness of breath on exertion, generalized weakness, easy fatigability, pallor, and gradual nail changes lasting about one month. He reported that he had been smoking cigarettes and marijuana for 40 years. He was not diabetic, had normal blood pressure, and had no known history of chronic liver or kidney disease. He did not mention any specific dietary restrictions. He also described poor hygiene practices while working on the farm, such as not consistently washing hands or fruits before eating.

Physical examination showed pallor and weight loss. Nail examination revealed characteristic koilonychia affecting multiple fingernails and toenails (Figure 1).

**Figure 1:**
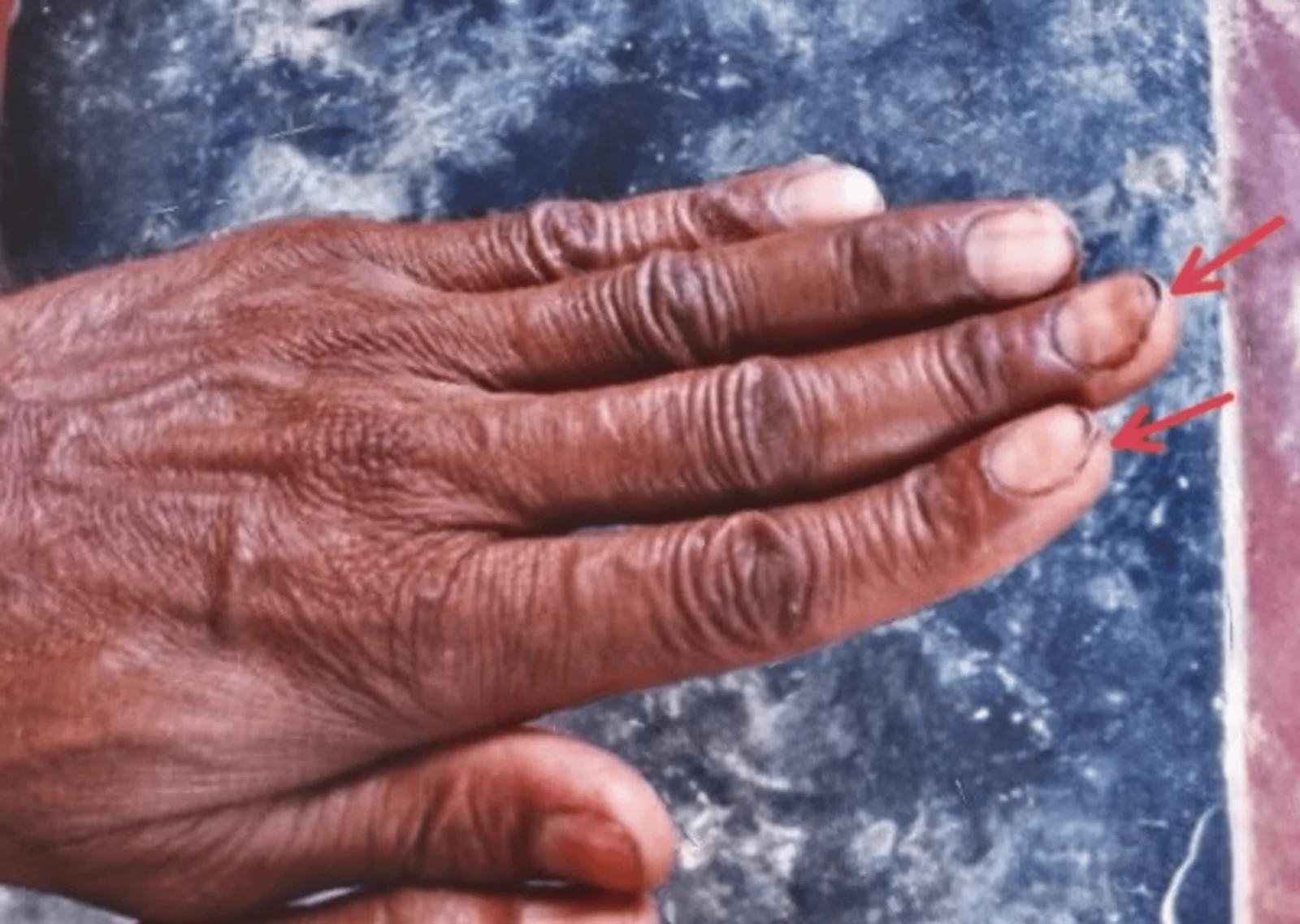
Koilonychia. Clinical photograph showing koilonychia with spoon-shaped nails, suggestive of chronic iron deficiency anemia.

Vital signs were within normal limits. Respiratory examination showed normal bilateral breath sounds without wheeze or rhonchi. Abdominal examination revealed no organomegaly, lump, or tenderness. Other systemic examinations were unremarkable.

Diagnostic assessment

Initial hematological tests showed severe anemia with microcytosis and hypochromia. Hemoglobin was 4.2 g/dL, red blood cell count was 3.1 × 10^6^/mm^3^, mean corpuscular volume was approximately 62 fL, and mean corpuscular hemoglobin was approximately 17 pg. Peripheral blood film showed microcytic hypochromic anemia. Iron studies indicated severe depletion of iron stores, with serum ferritin 1.92 ng/mL, serum iron 13.8 µg/dL, and total iron-binding capacity 409 µg/dL. The initial findings are summarized in Table 1.

**Table 1:** Initial laboratory and endoscopic investigation findings. HBsAg = hepatitis B surface antigen; HCV = hepatitis C virus; HIV = human immunodeficiency virus

Investigation	Finding	Reference values	Interpretation
Hemoglobin	4.2 g/dL	Male: 13–18 g/dL; female: 11.5–15.5 g/dL	Severe anemia
Red blood cell count	3.1 × 10^6^/mm^3^	Male: 4.5-5.5 × 10^6^/mm^3^; female: 3.8-4.8 × 10^6^/mm^3^	Reduced red blood cell count
Mean corpuscular volume	Approximately 62 fL	76–96 fL	Microcytosis
Mean corpuscular hemoglobin	Approximately 17 pg	27–32 pg	Hypochromia
Peripheral blood film	Microcytic hypochromic anemia	-	Consistent with iron deficiency anemia
Serum ferritin	1.92 ng/mL	8–388 ng/mL	Severe depletion of iron stores
Serum iron	13.8 µg/dL	Male: 80–180 µg/dL; female: 60–160 µg/dL; newborn: 100–250 µg/dL	Low serum iron
Total iron-binding capacity	409 µg/dL	Male: 291–430 µg/dL; female: 274–494 µg/dL	Elevated/upper range, supporting iron deficiency
Total white blood cell count	8,100/mm^3^	Adult: 4000–11000/mm^3^	Within normal range
Platelet count	374 × 10^3^/mm^3^	150–400 × 10^3^/mm^3^	Within normal range
HBsAg, anti-HCV, HIV	Negative	-	Viral screening negative
Upper gastrointestinal endoscopy	Motile duodenal worms with antral erosions	-	Duodenal ascariasis detected during anemia evaluation

Stool microscopy is an important initial investigation for suspected intestinal helminthiasis in endemic regions because it may detect ova or larvae and may help evaluate parasitic causes of gastrointestinal symptoms or anemia. In this patient, stool microscopy could not be performed at the time of initial evaluation, which is an important limitation. Because the patient was an elderly male with profound iron deficiency anemia and no clear dietary restriction or overt bleeding, upper gastrointestinal endoscopy was performed to evaluate a possible gastrointestinal source of anemia.

Upper gastrointestinal endoscopy revealed a normal esophagus, multiple antral erosions, and multiple long, cylindrical, creamy-white motile worms in the duodenal bulb and second part of the duodenum, consistent with duodenal ascariasis (Figure 2).

**Figure 2:**
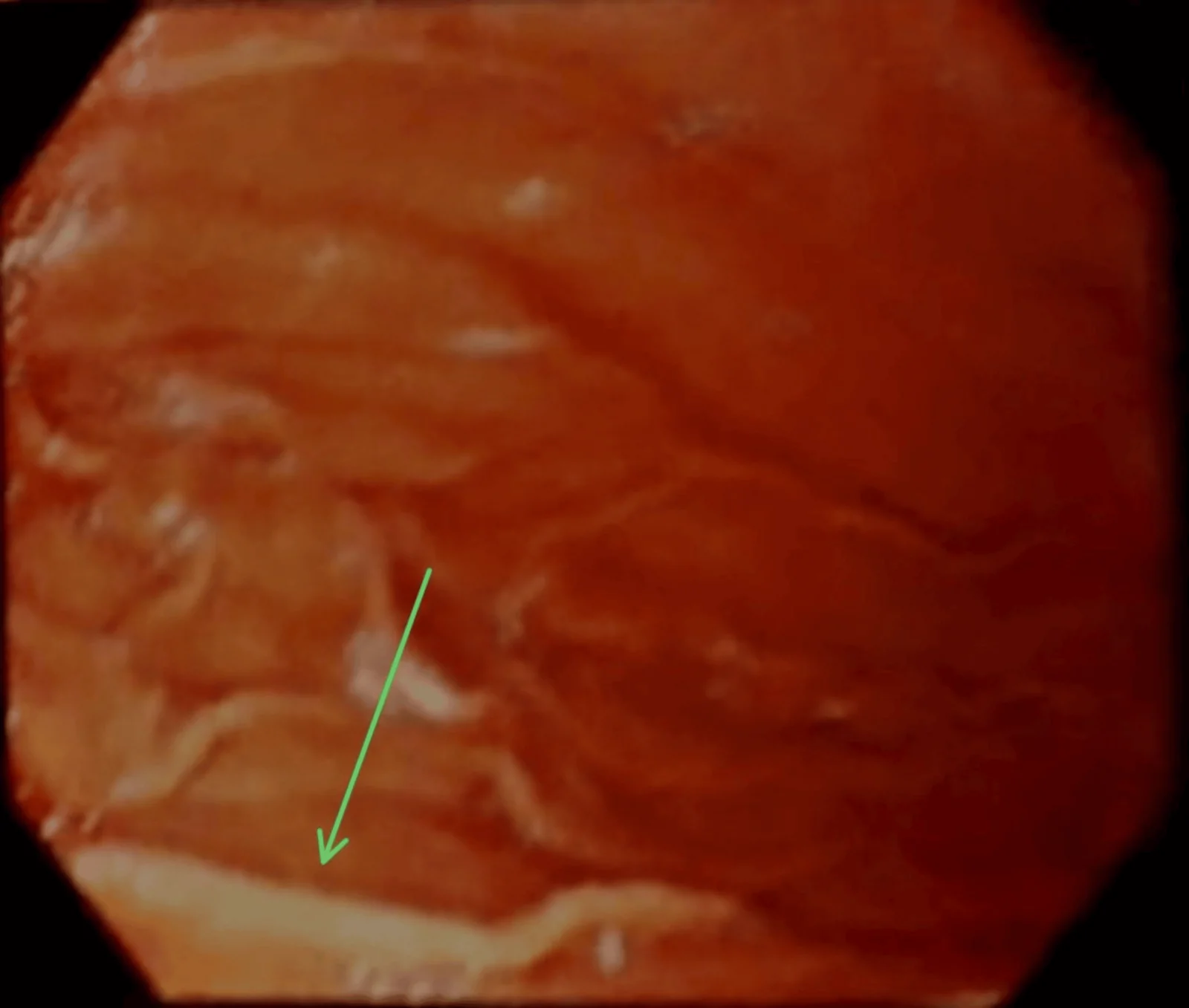
Duodenal ascariasis on upper gastrointestinal endoscopy. Upper gastrointestinal endoscopy showing long cylindrical whitish worms in the duodenum, consistent with duodenal ascariasis.

Chest radiography was normal. Liver enzymes, serum electrolytes, and serum creatinine were within normal limits. Screening for hepatitis B surface antigen, anti-hepatitis C virus antibody, and human immunodeficiency virus was negative. Overall, the findings supported severe iron deficiency anemia with endoscopically detected duodenal ascariasis as a potentially treatable associated condition in an endemic setting.

Therapeutic intervention and follow-up

The patient was given oral albendazole 400 mg as treatment for helminth infection, with a repeat dose scheduled one week later as per the managing team’s protocol. Throughout the hospitalization, four units of packed red blood cells were transfused based on clinical need to address severe symptomatic anemia. Following clinical improvement, he was discharged with recommendations for close monitoring. A combined regimen of oral carbonyl iron, folic acid, and zinc was prescribed to be taken twice daily over a three-month period. Table 2 provides a summary of the treatment plan.

**Table 2:** Treatment regimen used in the patient.

Treatment	Dose	Duration	Route
Albendazole	400 mg	Single dose, repeated after 1 week	Oral
Red cell concentrate	4 units	During hospital admission	Intravenous
Carbonyl iron + folic acid + zinc	50 mg + 0.50 mg + 61.80 mg	Twice daily for 3 months	Oral

At the three-month follow-up, the patient exhibited notable improvements in clinical status, blood parameters, and biochemical markers, including lessened dyspnea and fatigue and noticeable resolution of koilonychia. Hemoglobin levels rose to 14.8 g/dL, mean corpuscular volume to 92.5 fL, serum iron to 110 µg/dL, total iron-binding capacity to 314 µg/dL, and serum ferritin to 95.1 ng/mL. Table 3 shows the follow-up results.

**Table 3:** Three-month follow-up investigation findings.

Parameter	Follow-up finding	Reference value
Hemoglobin	14.8 g/dL	Male: 13–18 g/dL; female: 11.5–15.5 g/dL
Mean corpuscular volume	92.5 fL	76–96 fL
Mean corpuscular hemoglobin	31.0 pg	27–32 pg
Red blood cell count	4.77 × 10^6^/mm^3^	Male: 4.5–5.5 × 10^6^/mm^3^; female: 3.8–4.8 × 10^6^/mm^3^
Serum iron	110 µg/dL	Male: 80–180 µg/dL; female: 60–160 µg/dL; newborn: 100–250 µg/dL
Total iron-binding capacity	314 µg/dL	Male: 291–430 µg/dL; female: 274–494 µg/dL
Serum ferritin	95.1 ng/mL	8–388 ng/mL
Total white blood cell count	9,400/mm^3^	Adult: 4,000–11,000/mm^3^
Platelet count	230,000/mm^3^	150,000–400,000/mm^3^

## Discussion

*Ascaris lumbricoides* is a soil-transmitted helminth that remains prevalent in tropical and subtropical areas, particularly where sanitation and hygiene infrastructure are inadequate [2,3]. Infection arises from ingesting embryonated eggs present in contaminated soil, food, or water. Given the patient’s epidemiological profile, including agricultural work, rural exposure, and poor hand and food hygiene, parasitic infection was a relevant clinical consideration.

A key analytical challenge in such cases is establishing causality. Hookworm infection is a recognized cause of iron deficiency anemia due to chronic intestinal blood loss, whereas ascariasis typically leads to asymptomatic infection or nonspecific gastrointestinal symptoms and is a less direct cause of iron deficiency anemia [4,5,7]. Thus, the duodenal ascariasis in this patient should be viewed as an endoscopically confirmed associated condition that is potentially treatable, rather than definitive evidence that ascariasis alone caused the anemia.

In standard clinical practice, stool microscopy remains a primary initial diagnostic test when intestinal helminthiasis is suspected. Upper gastrointestinal endoscopy is not a first-line diagnostic tool for ascariasis. In this patient, endoscopy was performed due to severe iron deficiency anemia in an elderly male, where gastrointestinal conditions such as erosive disease, ulceration, malignancy, or other bleeding sources needed to be ruled out. The observation of motile worms in the duodenum was therefore an incidental endoscopic finding during anemia workup, not the main diagnostic approach for parasitic disease. This distinction is crucial to avoid implying that endoscopy should precede stool examination in suspected helminthiasis.

Koilonychia is traditionally linked to chronic iron deficiency anemia, but it is not exclusive to iron deficiency. It can also be seen in thyroid disease, repeated nail trauma, eating disorders, occupational exposure to petroleum products, celiac disease, cardiovascular disease, systemic lupus erythematosus, Raynaud phenomenon, and other conditions [6]. In this patient, koilonychia was assessed in the context of severe microcytic hypochromic anemia and markedly depleted iron stores. Therefore, koilonychia indicated chronic iron deficiency but did not, on its own, reveal the underlying cause.

Previous reports on helminth-associated iron deficiency anemia have predominantly highlighted hookworm infection, as hookworm directly causes chronic intestinal blood loss [8]. However, adult *Ascaris* infection has also been unexpectedly identified during evaluation of iron deficiency anemia, including via capsule endoscopy and double-balloon enteroscopy [9,10]. Compared to those reports, the distinctive aspects of this case are pronounced koilonychia, severe anemia with hemoglobin of 4.2 g/dL, and direct visualization of multiple motile worms in the duodenum during upper gastrointestinal endoscopy. This case is instructive because it shows how parasitic disease can be discovered during gastrointestinal evaluation of severe anemia in an endemic area, while also underscoring the need to avoid over-attributing causality when stool microscopy and lower gastrointestinal evaluation are incomplete.

The differential diagnosis for severe microcytic hypochromic anemia in a 60-year-old male includes chronic gastrointestinal blood loss, gastrointestinal malignancy, nutritional deficiency, malabsorption, parasitic infestation, thalassemia trait, and anemia of chronic disease. Very low ferritin, low serum iron, and elevated total iron-binding capacity strongly favored iron deficiency anemia over isolated thalassemia trait or anemia of chronic disease [1]. In South Asia, thalassemia trait may coexist with iron deficiency, and further evaluation may be warranted if microcytosis persists after iron repletion. In elderly male patients, gastrointestinal malignancy and occult gastrointestinal bleeding remain important considerations, particularly if anemia recurs or does not stay corrected after treatment.

Management in this case addressed both the severe anemia and the endoscopically detected helminthic infection. The patient received albendazole as anthelminthic therapy, red cell concentrate transfusion based on clinical status, and oral iron supplementation. The substantial improvement in hemoglobin, red cell indices, serum iron, ferritin, and symptoms at three months supports effective correction of iron deficiency. However, clinical improvement after combined deworming, transfusion, and iron therapy does not establish that ascariasis was the sole cause. Follow-up should include repeat complete blood count and iron studies, stool examination if feasible, reinforcement of hygiene and reinfection prevention, and further gastrointestinal evaluation if anemia persists or recurs.

This case has several limitations. Stool microscopy could not be performed at the time of initial evaluation; therefore, ova detection was not documented, and coexisting helminthic infection, particularly hookworm, could not be ruled out. Colonoscopy was also not performed during the initial evaluation, so lower gastrointestinal bleeding or malignancy cannot be entirely excluded. The patient improved clinically, hematologically, and biochemically after anthelminthic therapy, transfusion, and iron supplementation; however, because these therapies were administered together, the individual contribution of each intervention cannot be determined. As this is a single case report, a definitive causal relationship between ascariasis and severe iron deficiency anemia cannot be established. Therefore, the case should be interpreted as severe iron deficiency anemia with endoscopically detected duodenal ascariasis in an endemic setting, rather than proof that ascariasis was the sole cause.

## Conclusions

This case underscores the need for a thorough assessment of severe iron deficiency anemia accompanied by koilonychia in older male patients. In areas where parasitic infections are common, they should be included in the differential diagnosis; however, stool microscopy continues to be a key standard test, and other potential causes such as gastrointestinal bleeding, cancer, poor nutrition, and malabsorption also need to be investigated. The endoscopic view of duodenal ascariasis in this patient revealed a treatable coexisting issue, and the patient’s condition improved following treatment with anthelmintic drugs, blood transfusion, and iron supplementation.
